# Extended haplotypes at the complement factor H gene locus are associated with liver transplant rejection

**DOI:** 10.1097/HC9.0000000000000997

**Published:** 2026-07-15

**Authors:** Lianne M. Nieuwenhuis, Bao-Li Loza, Stefan P. Berger, Bernadien H. Jansen, Hans Blokzijl, Mohamed R. Daha, Rosa G.M. Lammerts, Avi Shaked, Sumeet Asrani, TransplantLines Investigators, Ton Lisman, Rinse K. Weersma, Eleonora A. M. Festen, Vincent E. de Meijer, Felix Poppelaars

**Affiliations:** 1 Department of Surgery, Section of Hepatopancreatobiliary Surgery and Liver Transplantation, University Medical Center Groningen, University of Groningen, Groningen, The Netherlands; 2 Department of Gastroenterology and Hepatology, University Medical Center Groningen, University of Groningen, Groningen, The Netherlands; 3 Department of Surgery, Penn Transplant Institute, Perelman School of Medicine, University of Pennsylvania, Philadelphia, Pennsylvania, USA.; 4 Department of Internal Medicine, Division of Nephrology, University Medical Center Groningen, University of Groningen, Groningen, The Netherlands; 5 Department of Nephrology, Leiden University Medical Center, University of Leiden, Leiden, The Netherlands; 6 Transplantation Immunology, Department of Laboratory Medicine, University Medical Center Groningen, University of Groningen, Groningen, The Netherlands; 7 Baylor University Medical Center, Dallas, Texas, USA

**Keywords:** acute cellular rejection, genetics, immunology, liver disease, liver transplantation

## Abstract

**Background:**

Acute T-cell–mediated rejection (ACR) remains an important complication after liver transplantation. Polymorphisms in the factor H gene (*CFH*) and factor H-related protein genes (*CFHRs*) affect the risk of multiple complement-related diseases. Given the complement system’s key role in alloimmunity, we hypothesized that variants in *CFH* and *CFHRs* associate with ACR risk after liver transplantation.

**Methods:**

In 689 donor–recipient pairs, the deletion of *CFHR1* and *CFHR3* (*CFHR3,1Δ*), a low-expression variant of *CFHR2*, and *CFH* haplotypes were extracted from both donor and recipient genotypes following appropriate imputation and quality control steps.

**Results:**

Kaplan–Meier survival curves and multivariable Cox regression analysis showed increased ACR incidence after transplantation of donor livers carrying 2 copies of the *CFHR3,1Δ* (log-rank *p*=0.03, HR: 2.76, 95% CI: 1.18–6.45) or the related *CFH*-H4a haplotype (log-rank *p*=0.03, HR: 1.65, 95% CI: 1.04–2.63). In contrast, a lower ACR incidence was observed after transplanting donor livers with a low-expressing *CFHR2* variant (log-rank *p*=0.04, HR: 0.66, 95% CI: 0.45–0.96) or the related *CFH*-H2 haplotype (log-rank *p*=0.01, HR: 0.58, 95% CI: 0.37–0.92). When analyzing recipient genotypes, only *CFHR3,1Δ* was associated with ACR (log-rank: *p*=0.01, HR: 2.55, 95% CI: 1.15–5.65).

**Conclusions:**

Common functional variants in the *CFH*–*CFHR* region are associated with rejection risk after liver transplantation, highlighting a previously overlooked relevance of the complement system in liver transplantation.

## Graphical abstract

**Figure:**
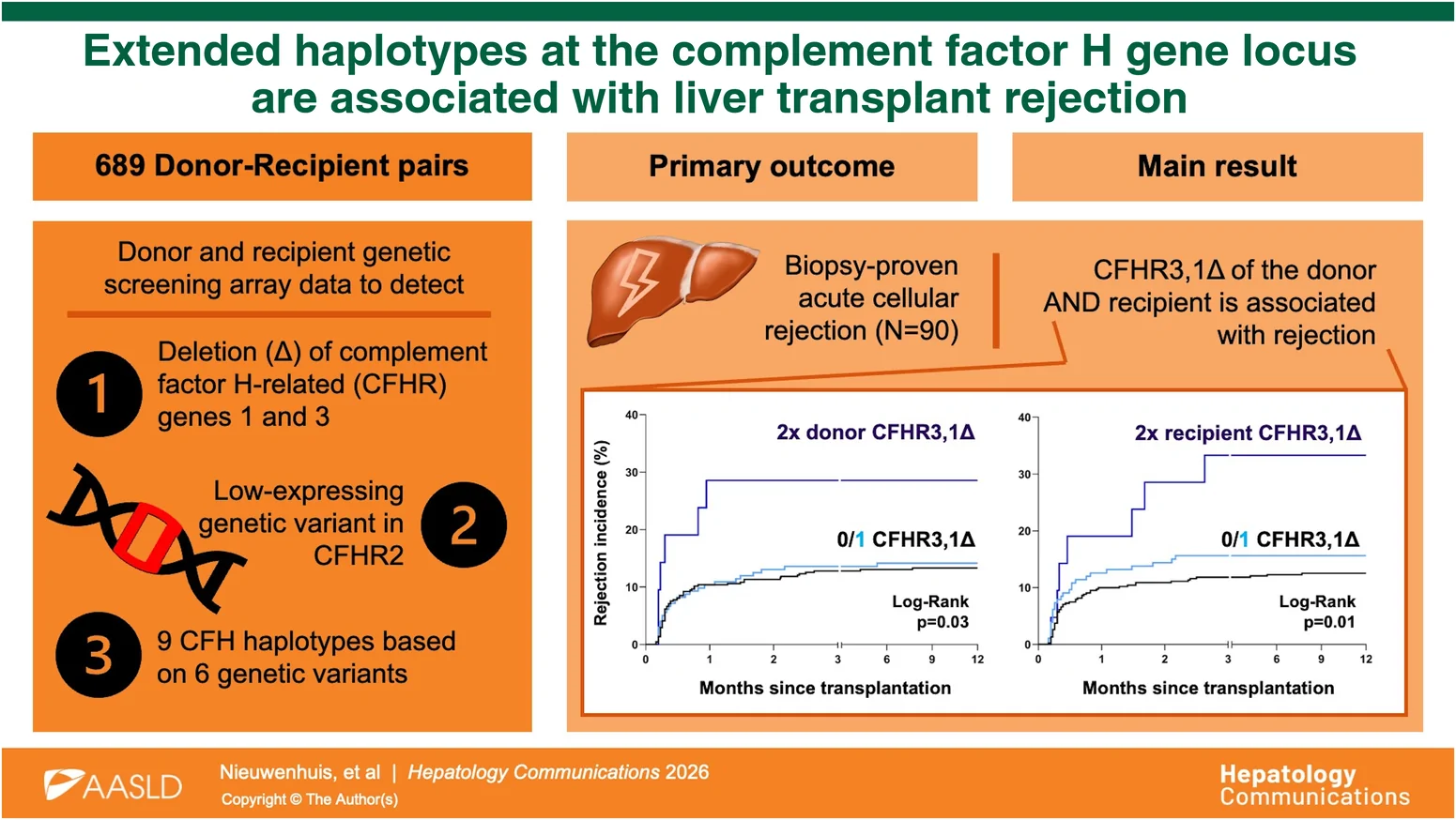

## INTRODUCTION

Despite the liver graft’s inherent tolerogenic properties, 15%–25% of liver transplant recipients develop acute T-cell–mediated rejection (ACR), often necessitating intensified immunosuppression that increases the risk of infections and toxicity.^1^ Risk factors for ACR include autoimmune-based liver diseases, non-HLA (human leukocyte antigen) mismatch, younger recipients, older donors, prolonged cold ischemia time, and low tacrolimus trough levels.^2^^,^^3^ However, the biological mechanisms driving ACR remain poorly understood. A deeper understanding of these mechanisms could improve ACR risk assessment and patient-specific therapy, and as a result, reduce unnecessary treatment.

The importance of the complement system in transplantation, especially in kidney transplantation, has been extensively described.^4^^,^^5^ As a key component of innate immunity, this system facilitates opsonization, chemotaxis, immune clearance, and drives and shapes B-cell and T-cell responses.^6^ In liver transplantation, complement activation occurs before,^5^ during,^7^ and after transplantation,^8^ driven by donor conditions, ischemia-reperfusion, and allosensitization.^4^^,^^5^ Furthermore, animal studies have shown that complement-targeting therapeutics can reduce allograft injury during liver transplantation, although clinical trials remain limited.^9^^,^^10^

The complement system is tightly regulated to prevent unwanted activation and subsequent harm.^11^ Factor H, a key complement regulator encoded by *CFH* on chromosome 1 and produced by the liver, controls complement activation in circulation and on cell surfaces.^12^ Genetic variations in *CFH* greatly impact the plasma levels and functionality of factor H.^12^ Specific *CFH* haplotypes, formed by combinations of common single-nucleotide polymorphisms (SNPs), have been associated with the risk of infectious and inflammatory diseases.^12^^,^^13^

In addition to factor H, 5 factor H-related proteins (FHRs) are encoded in tandem in the *CFH* locus; *CFHR3*, *CFHR1*, *CFHR4*, *CFHR2*, and *CFHR5* (Supplemental Figure S1A, http://links.lww.com/HC9/C389).^12^ Studies suggest that these FHRs modulate complement activation by competing with factor H for ligand binding, thereby potentially antagonizing or dysregulating complement control.^12^ Recently, genetic studies have identified associations between *CFHR* variants and multiple pathologies.^12^ Among these, the combined deletion of *CFHR3* and *CFHR1* (*CFHR3,1Δ*) is the most widely investigated, exhibiting a variable allele frequency between 0% and 55% across different geographical locations.^14^ Large genome-wide association studies have linked this common deletion to several immune-mediated diseases.^13^^,^^15^^,^^16^ In kidney transplantation, the *CFHR3,1Δ* has been associated with increased risk of ACR.^17^ Another notable *CFHR* variant, rs4085749, located within *CFHR2*, disrupts normal splicing and leads to near-null levels of FHR-2.^18^^,^^19^ GWAS have consistently shown that this rs4085749 SNP is associated with reduced circulating levels of FHR-2.^18^^–^^23^ Although the low-expressing *CFHR2* variant itself has only been associated with age-related macular degeneration (AMD), haplotypes carrying this variant have been associated with a reduced risk of atypical hemolytic uremic syndrome (aHUS) and C3 glomerulopathy.^24^^,^^25^

As the impact of polymorphisms in the *CFH–* *CFHRs* region remains unexplored in liver transplantation, our study investigates the relationship between these common genetic variations and ACR. Specifically, we analyzed the impact of the *CFHR3,1Δ*, the low-expressing *CFHR2* variant (rs4085749), and *CFH* haplotypes in liver transplant donor–recipient pairs on the incidence of ACR.

## METHODS

### Study design and patients

We included all liver transplantations at the University Medical Center Groningen (Netherlands, 1993–2019), excluding cases without genotype screening array data for either donor or recipient. Replication cohorts included liver transplants from Baylor University Medical Center (United States, 1998–2010; Simmons Transplant Institute biorepository^26^) and Penn Medicine Transplant Institute (United States, 2012–2017; BioTIP^27^). Donor–recipient characteristics were retrieved from institutional databases, and postoperative care, including immunosuppression, followed center-specific protocols as described previously.^26^^–^^28^

### Ethical approval

The Dutch cohort was conducted within the TransplantLines cohort study,^29^ which was approved by the institutional research board (METc 2014/077). The replication cohorts were approved by Institutional Review Boards at the respective institutions (Baylor: FWA 00004415/00003358, protocol 013-065; UPenn: FWA 00004028, protocol 820091). All participants provided informed consent prior to transplantation. All protocols followed the Declaration of Helsinki and the Declaration of Istanbul on Organ Trafficking and Transplant Tourism. STrengthening the REporting of Genetic Association studies (STREGA) guidelines were adhered to.^30^

### Primary outcome

Clinically relevant ACR was defined as Banff grade 2 or 3 biopsy-proven rejection within 1-year post-transplant, which was evaluated by experienced transplant pathologists. Recipients with clinically suspected rejection (without biopsy) or grade 1 biopsy-proven ACR were excluded from all analyses. The decision to focus on grade 2 or 3 rejection was guided by 2 considerations. First, these rejection grades are clinically significant, typically necessitating intensified therapy such as high-dose prednisolone. Second, because biopsies were performed only when clinically indicated, we could not reliably capture all grade 1 rejections, which often present with minimal or no symptoms and may therefore go undetected.

### Extended *CFH* haplotype analysis

Donor and recipient DNA were genotyped, quality-controlled, and imputed using the TOPMed panel. *CFHR3,1Δ* was inferred using the tag SNP rs6677604. In addition, we included rs4085749, a nonsense variant in *CFHR2*. *CFH* haplotypes were determined using 6 SNPs within *CFH*, phased with SHAPEIT. The full methodology, including details on HLA imputation and mismatch calculation, and replication analyses, is described in Supplemental Text 1, http://links.lww.com/HC9/C394.

### Statistical analyses

Baseline characteristics were presented as median (IQR) for continuous data and percentage [n (%)] for nominal data. Potential clinical confounders were identified by comparing donor, recipient, and surgical characteristics between ACR and non-ACR groups using Mann–Whitney or Chi-square tests (Fisher’s exact test for groups <N=5). Variables with >80% valid data and *p*<0.1 were included in multivariable Cox regression analyses.

Associations with ACR for donor and recipient *CFHR* genotypes and *CFH* haplotypes were analyzed using Kaplan–Meier survival curves with log-rank tests, followed by univariable and multivariable Cox regression. Due to low homozygous *CFHR3,1Δ* frequency, confounder correction was done across models to limit simultaneous variable adjustment: (1) baseline, (2) recipient-adjusted, (3) surgical-adjusted, and (4) fully adjusted. A priori and post hoc power analyses were performed to evaluate the adequacy of the sample size. Based on expected allele frequencies of 19% for *CFHR3,1Δ* and 25% for *CFHR2*, and an assumed effect size of 1.8, the a prior power analysis indicated that the available cohort (N=689) exceeded requirements for 80% power. The post hoc power analyses were performed using the powerSurvEpi package in R. This approach allows for power calculations in survival analyses while accounting for the correlation between the risk variable (exposure) and potential confounders. Two-sided *p*-values <0.05 were considered significant. Analyses were performed using PLINK (v1.9), SPSS (v28), and R (v4.3).

## RESULTS

### Patient characteristics and determinants of acute rejection

Of the 798 donor–recipient liver transplant pairs with genotype data, 109 pairs were excluded due to biochemically diagnosed rejection or grade 1 biopsy-proven rejection. The characteristics of the remaining 689 pairs are shown in Table 1. During a median follow-up of 10.3 years (IQR: 5.1–17.0), ACR developed in 90 liver transplant recipients (13%) after a median of 10 days (IQR: 7–25). In univariable analyses, the indications primary sclerosing cholangitis, primary biliary cholangitis, or autoimmune hepatitis, recipient cytomegalovirus (CMV) status, cold ischemia time, transplantation era, and donor type were associated with ACR (*p*<0.10, Table 1). Further analysis revealed a strong correlation (*r* ^2^: 0.42, *p*<0.001) between donor type and transplant era, defined as time in years from each transplantation to a fixed date identical for all transplantations (September 7, 2022), indicating that the association with donor type was confounded by differences in transplant era. Consequently, donor type was not included in the multivariable analyses to avoid multicollinearity, and time since transplantation (transplant era) was included instead, along with immune-mediated transplant indications, recipient CMV status, and cold ischemia time.

**TABLE 1:** Demographics of all donor–recipient pairs

	Rejection (N=90)		No rejection (N=599)				
	N/median	%/IQR	N/median	%/IQR	*p* ^a^	HR^b^	*p* ^b^
Recipient age, years	45	22–55	46	15–58	0.57		
Pediatric recipients	19	21.1	164	27.4	0.14		
Recipient sex, male	47	52.2	344	57.4	0.35		
Recipient BMI, kg/m^2^	23.3	19.5–26.0	23.3	19.0–27.0	0.70		
Primary disease, yes							
Acute liver failure	2	2.2	30	5.0	0.42		
PBC/AIH	15	16.7	58	9.6	**0.045**	1.80	**0.04**
PSC	25	27.8	77	12.9	**<0.001**	2.27	**<0.001**
Metabolic	9	10.0	86	14.4	0.26		
Congenital biliary disease	10	11.1	98	16.4	0.20		
Alcoholic liver disease	6	6.7	61	10.2	0.29		
Hepatitis	13	14.4	56	9.3	0.13		
Viral and drug-induced							
MASLD	4	4.4	36	6.0	0.55		
Other	6	6.7	97	16.2	0.02	0.41	**0.03**
Recipient CMV status at transplantation, positive	58	66	300	50	0.01	1.71	**0.02**
Donor age, years	43	32–53	46	30–56	0.52		
Donor sex, male	41	45.6	321	53.6	0.16		
Donor BMI, kg/m^2^	24	22–26	24	22–26	0.44		
Donor type, yes							
DBD	81	90.0	468	78.1	0.01	2.57	**0.01**
DCD	8	8.9	101	16.9	0.05	0.48	**0.04**
Living	1	1.1	30	5.0	0.11		
Cold ischemia time, min	516	430–602	463	382–585	0.05	1.001	0.08
Recipient warm ischemia time, min	47	38–54	45	37–54	0.14		
Graft type, whole	79	87.8	484	80.8	0.11		
Transplantation era (time since transplantation in years)	13.2	8.5–20.5	20.7	16.7–22.5	**<0.001**	1.07	**<0.001**

*Note:* Categorical variables are presented as numbers and percent (%), continuous variables are shown in median and interquartile range.

^a^ *p*-values were calculated using Mann–Whitney for continuous variables and Chi-square or Fisher's exact test (N<5) for categorical variables.

^b^ *p*-values and hazard ratios (HR) were calculated using univariable Cox regression analysis for 1-year ACR.

Abbreviations: ACR, acute cellular rejection; AIH, autoimmune hepatitis; BMI, body mass index; CMV, cytomegalovirus; DBD, donation after brain death; DCD, donation after cardiac death; HR, hazard ratio; MASLD, metabolic dysfunction–associated steatotic liver disease; PBC, primary biliary cholangitis; PSC, primary sclerosing cholangitis.

### The association of the *CFHR3,1Δ* with acute rejection

We first investigated whether *CFHR3,1Δ* affects ACR risk after liver transplantation. The *CFHR3,1Δ* tag SNP (rs6677604) had a minor allele frequency (MAF) of 18% in donors and 17% in recipients, aligning with the general European population’s MAF of 19%.^31^ Genotype frequencies in donors (GG, 67%; GA, 30%; AA, 3%) and recipients (GG, 69%; GA, 28%; AA, 3%) did not differ significantly (*p*=0.70). Post hoc analyses estimated that the sample size provided a power of 75% for donor *CFHR3,1Δ* and 65% for recipient *CFHR3,1Δ* under an additive model. Kaplan–Meier analysis revealed that transplanted livers from donors homozygous for the *CFHR3,1Δ* had significantly higher rates of ACR (Figure 1A, log-rank *p*=0.03). One year post-transplantation, livers from homozygous *CFHR3,1Δ* donors had an ACR incidence of 27%, compared with 12% in non-*CFHR3,1Δ* carriers. Surprisingly, Kaplan–Meier analysis also demonstrated that homozygosity for the *CFHR3,1Δ* in the recipient genome was associated with increased ACR incidence (Figure 1B, log-rank *p*=0.01). At 1-year post-transplantation, ACR incidence was 32% in recipients homozygous for the *CFHR3,1Δ*, compared with 12% in non-*CFHR3,1Δ* carriers. To exclude transplant era effects, a subgroup analysis was restricted to patients transplanted after April 9th, 1996, when tacrolimus was introduced. This ensured the cohort reflected modern transplant practice while retaining adequate statistical power. Kaplan–Meier analysis in this subgroup confirmed the full cohort findings: donor and recipient *CFHR3,1Δ* remained associated with higher ACR risk (*p*=0.04 and *p*<0.001, respectively; Supplemental Figures S2A, B, http://links.lww.com/HC9/C389). To further investigate the impact of the combined presence of the *CFHR3,1Δ* in donor–recipient pairs on ACR, we categorized transplant pairs into 4 groups according to the presence or absence of *CFHR3,1Δ* in both the donor and the recipient. Kaplan–Meier survival analysis showed significant differences in ACR rates among these 4 groups (Figure 1C, log-rank *p*=0.01). Donor–recipient pairs both carrying the *CFHR3,1Δ* (AA/AA or GA/AA) had the highest ACR incidence (33%), while those without the *CFHR3,1Δ* (GG/GG) had the lowest incidence (12%).

**FIGURE 1:**
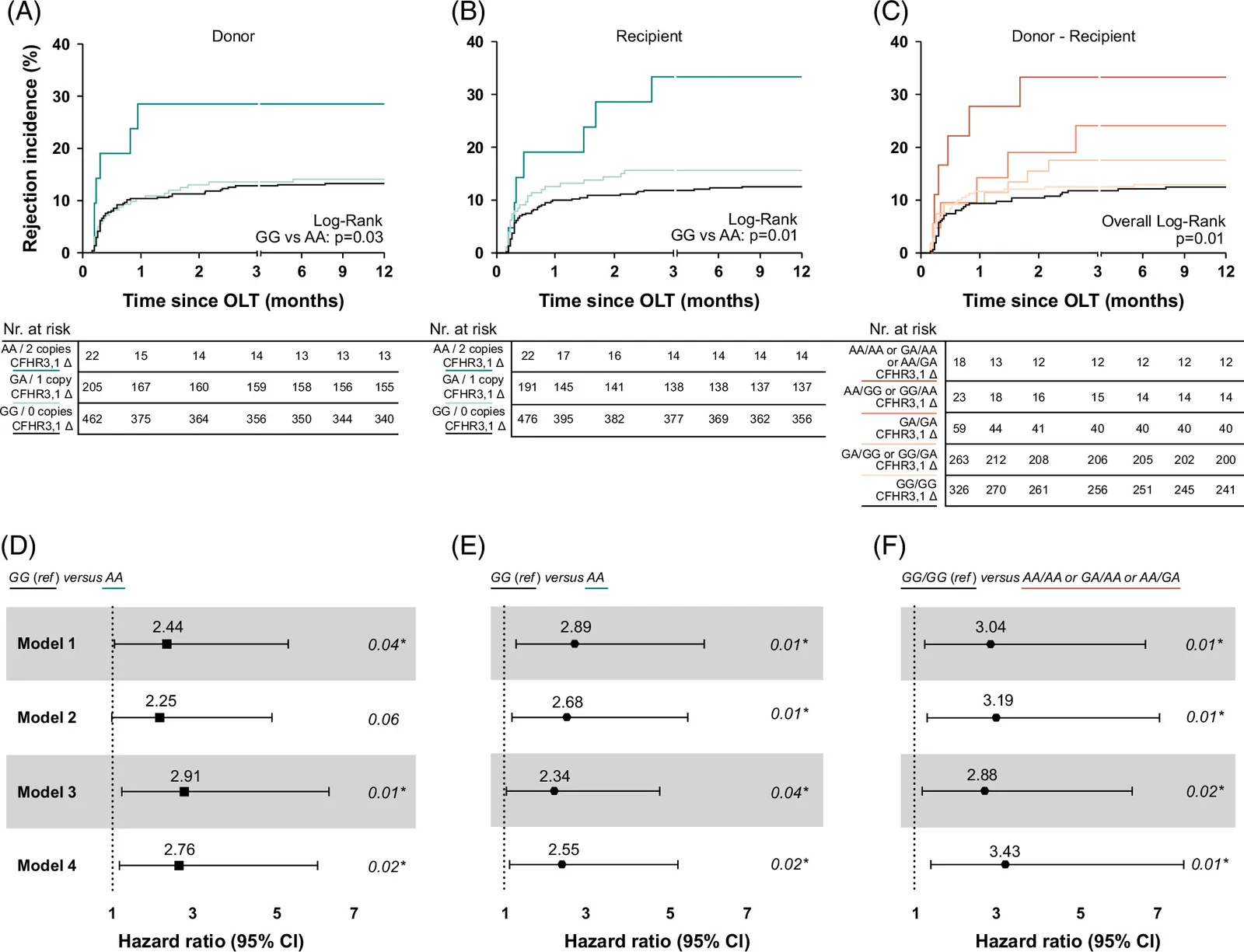
Kaplan–Meier cumulative risk curves and forest plots depicting increased ACR risk for homozygous *CFHR3,1Δ* genotypes in the donor and recipient. Kaplan–Meier cumulative risk curves, with log-rank testing, comparing ACR incidence between differing donor (A), recipient (B), and donor–recipient combined (C) *CFHR3,1Δ* genotypes. Forest plots depict univariable and multivariable Cox regression results for donor (D), recipient (E), and donor–recipient combined (F) *CFHR3,1Δ* genotypes. Model 1 depicts univariable Cox regression results. The *CFHR3,1Δ* genotypes were corrected for recipient-related confounders (immune-mediated transplant indications, recipient CMV status) in Model 2, for surgical-related confounders [cold ischemia time, time since transplantation (transplantation era)] in Model 3, and for all possible confounders, also called the “full” model, in Model 4. Abbreviations: ACR, acute cellular rejection; *CFHR3,1Δ*, deletion of *CFHR3* and *CFHR1*; CMV, cytomegalovirus; OLT, orthotopic liver transplantation.

Multivariable Cox regression analyses demonstrated that donor *CFHR3,1Δ* (Full model (4): HR: 2.76, 95% CI: 1.18–6.45, *p*=0.02; Figure 1D), recipient *CFHR3,1Δ* (Full model (4): HR: 2.55, 95% CI: 1.15–5.65, *p*=0.02; Figure 1E), and their combined presence (Full model (4): HR: 3.43, 95% CI: 1.44–8.20, *p*=0.01; Figure 1F) remained associated with ACR after adjusting for confounders. All models, except model 2 in the donor *CFHR3,1Δ* analysis (adjusted only for recipient factors), showed consistent and significant associations. Each additional *CFHR3,1Δ* copy (ie, GG to GA, or GA to AA) in either the donor or recipient increased ACR risk, with an HR of 1.36 per copy (95% CI: 1.06–1.74, *p*=0.02). Given the inconclusive literature on HLA mismatch and ACR in liver transplantation, we also performed a sensitivity analysis for this, which showed that HLA mismatch was not associated with ACR (HR: 1.01; 95% CI: 0.996–1.02; *p*=0.18), and did not alter the associations between the *CFHR3,1Δ* and ACR (Supplemental Table S3, http://links.lww.com/HC9/C392). Altogether, these findings indicate that the absence of *CFHR1* and *CFHR3* in the donor and/or recipient is associated with increased rejection risk post-liver transplantation.

### The association of a near-null allele in CFHR2 with acute rejection

Subsequently, we examined whether ACR risk was affected by another common *CFHR* deficiency, a low-expressing *CFHR2* variant (rs4085749). The MAF of this variant was 26% in donors and 25% in recipients, aligning with the general European population’s MAF of 25%.^31^ Furthermore, the genotype frequencies in donors (CC, 55%; CT, 39%; TT, 7%) and recipients (CC, 57%; CT, 36%; TT, 7%) did not differ significantly (*p*=0.67). Post hoc analyses estimated 96% power for donor *CFHR2* under an additive model, demonstrating the adequacy of the sample size. Kaplan–Meier analysis demonstrated that transplanted livers from donors with the low-expressing *CFHR2* variant had significantly lower rates of ACR (log-rank *p*=0.04; Figure 2A), showing a dosage effect for each additional minor allele. Furthermore, Kaplan–Meier analysis in the subgroup reflecting modern transplant practice confirmed the full cohort findings: donor *CFHR2* showed a dose-dependent effect (*p*=0.03; Supplemental Figure S2C, http://links.lww.com/HC9/C389).

**FIGURE 2:**
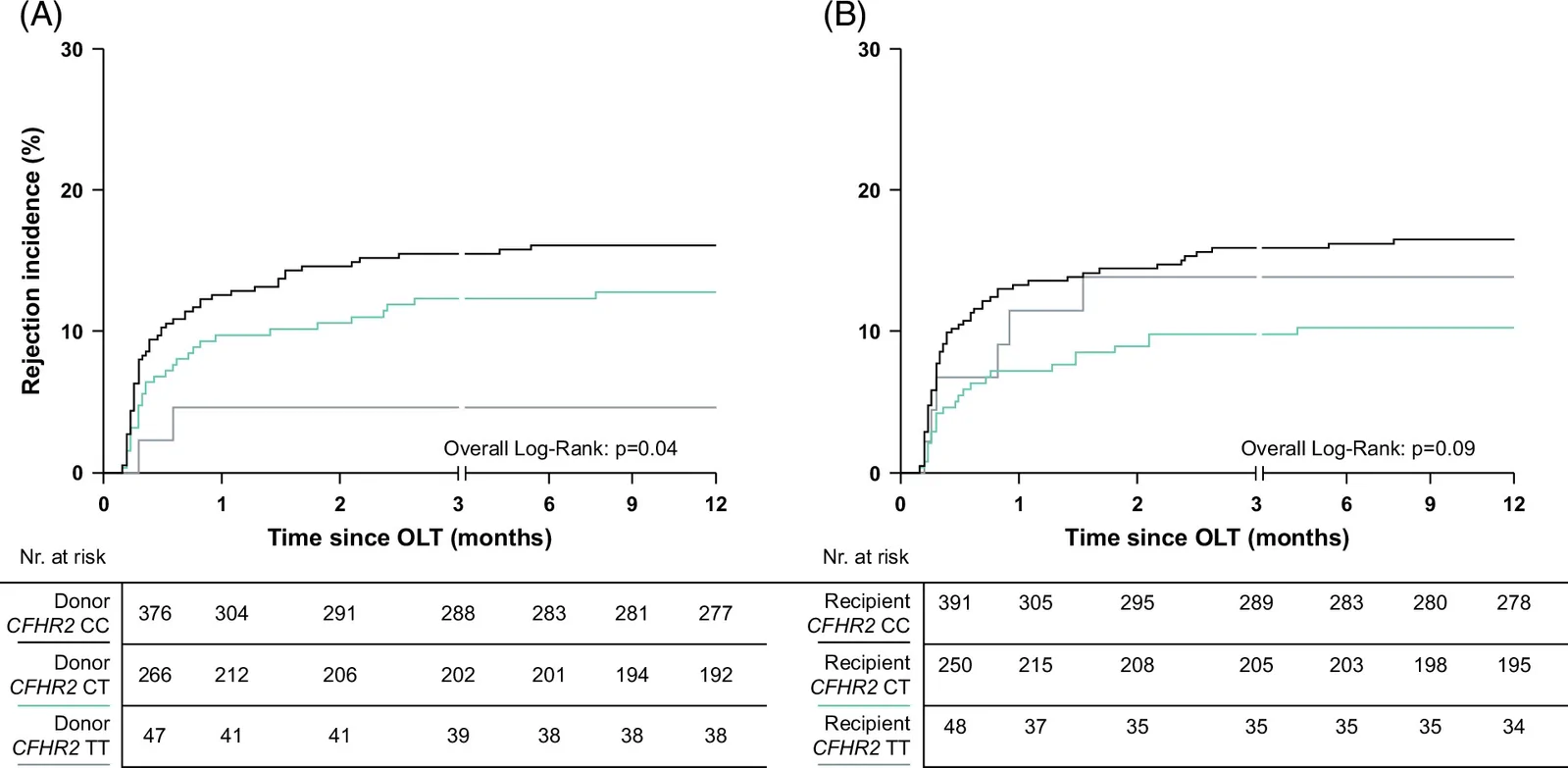
Kaplan–Meier cumulative risk curves depicting a protective effect on ACR incidence for the *CFHR2* low-expressing variant in the donor. Kaplan–Meier cumulative risk curves, with log-rank testing, comparing ACR incidence between differing donor (A) and recipient (B) *CFHR2* genotypes. Abbreviations: ACR, acute cellular rejection; OLT, orthotopic liver transplantation.

The association remained significant in univariable (HR: 0.68, 95% CI: 0.47–0.98, *p*=0.04) and multivariable (HR: 0.66, 95% CI: 0.45–0.96, *p*=0.03) Cox regression analyses, where we analyzed the *CFHR2* variant as an ordinal variable due to the observed dosage effect. Using the same approach as for the *CFHR3,1Δ* analysis, adjustment for HLA mismatch did not affect the association between the *CFHR2* variant and ACR (Supplemental Table S3, http://links.lww.com/HC9/C392). No significant difference in ACR was observed among the recipients’ *CFHR2* genotypes (log-rank *p*=0.09; Figure 2B). Overall, these results suggest that a low-expressing *CFHR2* variant in the donor is associated with decreased liver transplant rejection risk.

### The association between CFH haplotypes and acute rejection

As the *CFHR3,1Δ* and *CFHR2* variants are related to distinct *CFH* haplotypes,^13^^,^^18^ we sought to determine whether rejection risk was driven by the respective *CFH* haplotypes of these *CFHR* variants. *CFH* haplotype frequencies (Supplemental Figure S1C, http://links.lww.com/HC9/C389) and genotypes of the individual SNPs (Supplemental Table S1, http://links.lww.com/HC9/C390) were similar between liver transplant donors and recipients. We found that the *CFHR3,1Δ* was highly linked to the *CFH* H4a haplotype (*r* ^2^ donor: 0.67 and *r* ^2^ recipient: 0.68, both *p*<0.001), while the *CFHR2* variant strongly aligned with the *CFH* H2 haplotype (*r* ^2^ donor: 0.79 and *r* ^2^ recipient: 0.78, both *p*<0.001). Among *CFH* H4a-positive donors and recipients, 96% and 94% had the *CFHR3,1Δ*, compared with only 17% and 16% of donors and recipients without the *CFH* H4a haplotype. Similarly, 94% of both the *CFH* H2-positive donors and recipients carried the *CFHR2* low-expressing variant, compared with just 10% and 9% of donors and recipients without the *CFH* H2 haplotype. Supplemental Figure S3, http://links.lww.com/HC9/C389, shows the frequencies of the different combinations of the *CFH* H2 and *CFH* H4a haplotypes with the low-expressing *CFHR2* variant and *CFHR3,1Δ* in donors and recipients.

We then investigated the association between *CFH* haplotypes and ACR (Table 2) and classified individuals into haplotypes by the presence of at least one copy of the respective haplotype. *CFH* haplotypes 5–8 were combined into a single group, as their individual frequencies were <5%. In univariable analyses, donor *CFH* H2 haplotype was associated with a lower risk of ACR (HR: 0.62, 95% CI: 0.39–0.96, *p*=0.03), while donor *CFH* H4a haplotype was associated with a higher ACR risk (HR: 1.62, 95% CI: 1.03–2.56, *p*=0.04). When corrected for potential confounders in multivariable analyses, donor *CFH* H2 haplotype (HR: 0.58, 95% CI: 0.37–0.92, *p*=0.02) and donor *CFH* H4a haplotype (HR: 1.65, 95% CI: 1.04–2.63, *p*=0.04) remained associated with ACR. As expected, recipient *CFH* haplotypes were not associated with ACR risk (Supplemental Table S2, http://links.lww.com/HC9/C391).

**TABLE 2:** Association between complement factor H haplotypes in the donor and ACR

	Univariable			Multivariable		
90 rejection versus 599 controls	HR	95% CI	*p*	HR	95% CI	*p*
H1, yes	1.40	0.91–2.14	0.12	—	—	—
H2, yes	0.62	0.39–0.96	**0.03**	0.58	0.37–0.92	**0.02**
H3, yes	0.91	0.57–1.48	0.71	—	—	—
H4a, yes	1.62	1.03–2.56	**0.04**	1.65	1.04–2.63	**0.04**
H4b, yes	1.26	0.71–2.22	0.44	—	—	—
H5–8, yes	0.93	0.51–1.71	0.82	—	—	—

*Note:* Univariable and multivariable Cox regression analyses were performed with ACR as a binary outcome (presence or absence). For each individual haplotype, we set the reference as the absence (zero copies) of that haplotype, with the presence of a haplotype meaning at least one copy of that haplotype. Only haplotypes that showed a significant association in the univariable analyses were included in the multivariable analyses. Data are presented as hazard ratios with confidence ratios and corresponding *p*-values. In the multivariable analysis, we have corrected for immune-mediated transplant indications (PBC, PSC, AIH), CIT, recipient CMV status at OLT, and time since transplantation.

Abbreviations: ACR, acute cellular rejection; AIH, autoimmune hepatitis; CI, confidence interval; CIT, cold ischemia time; CMV, cytomegalovirus; HR, hazard ratio; OLT, orthotopic liver transplantation; PBC, primary biliary cholangitis; PSC, primary sclerosing cholangitis.

Mirroring the association of *CFHR3,1Δ* with ACR, Kaplan–Meier analysis demonstrated significantly higher ACR incidence for donor livers with the *CFH* H4a haplotype (Figure 3A, log-rank *p*=0.04). Due to the small number of homozygous H4a donors (N=7), heterozygous and homozygous H4a donors were combined in this analysis. One year post-transplantation, ACR incidence was 19% in donor livers carrying the *CFH* H4a haplotype and 12% in donor livers without the haplotype. In line with the association of the low-expressing *CFHR2* variant with ACR, Kaplan–-Meier analyses showed that donor livers with the *CFH* H2 haplotype had a significantly lower incidence of ACR (Figure 3B, log-rank *p*=0.01). One year post-transplant, ACR incidence was 4% in donor livers homozygous for the *CFH* H2 haplotype, 11% in the heterozygous group, and 16% in donor livers without the haplotype. Altogether, we found that specific *CFH* haplotypes in the donor graft were associated with ACR risk after liver transplantation.

**FIGURE 3:**
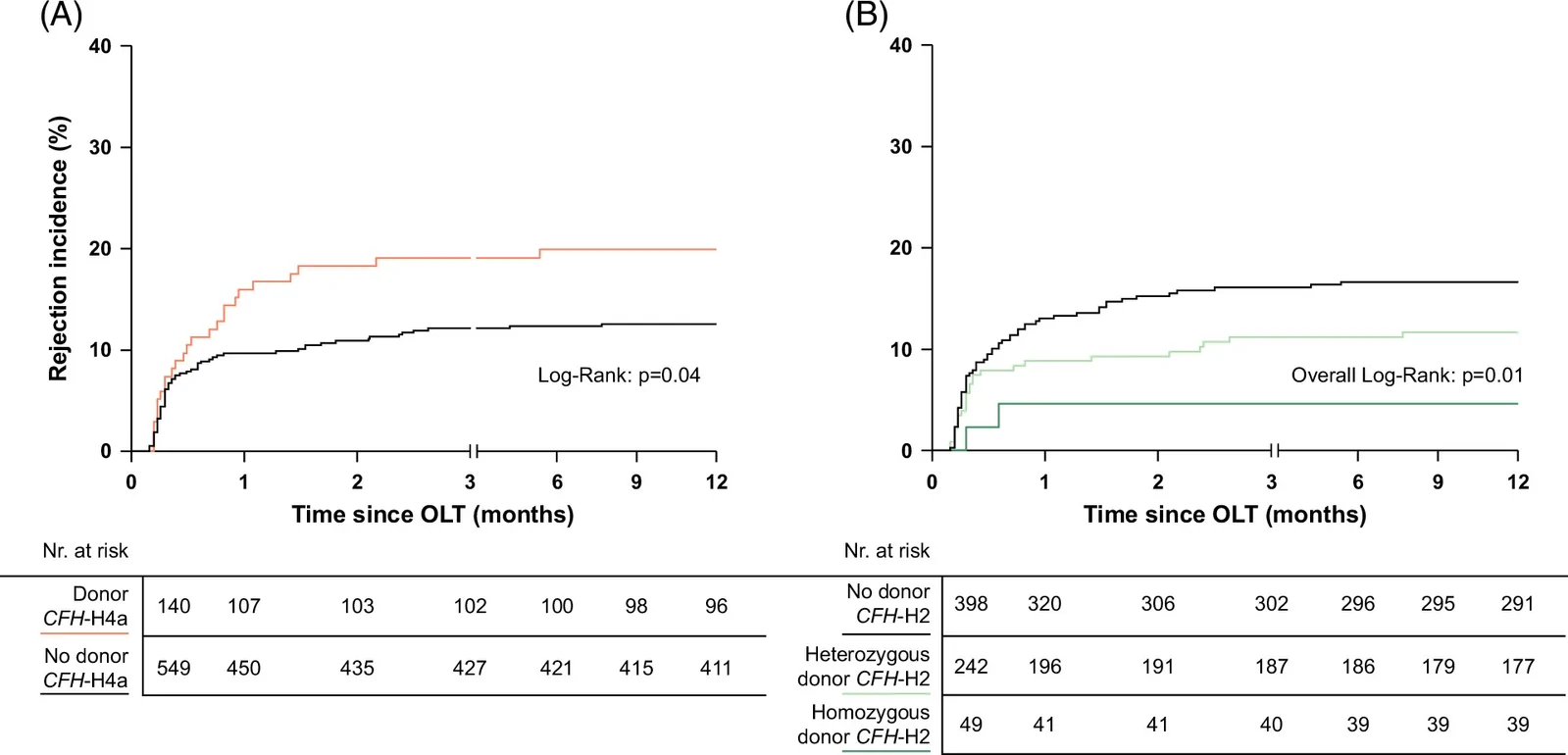
Kaplan–Meier cumulative risk curves depicting associations with ACR for *CFH* H2 and *CFH* H4a haplotypes. Kaplan–Meier cumulative risk curves, with log-rank testing, comparing ACR incidence between differing donor *CFH* H2 (A) and *CFH* H4a (B) haplotypes*. CFH* H4a heterozygous and homozygous donors were combined into one group due to low numbers in the homozygous *CFH* H4a group. Abbreviations: ACR, acute cellular rejection; *CFH*, complement factor H; OLT, orthotopic liver transplantation.

### Dissection of the associations of *CFHR* variants and *CFH* haplotypes with acute rejection

To dissect the respective effect of *CFHR* variants and *CFH* haplotypes on ACR risk, we conducted subgroup analyses to evaluate the association between *CFHR* variants and ACR within their corresponding *CFH* haplotypes. Among donor livers with one copy of the *CFH* H4a haplotype, Kaplan–Meier analysis showed a higher ACR incidence in those homozygous for the *CFHR3,1Δ* compared with *CFHR3,1Δ* heterozygotes (Figure 4A, log-rank *p*<0.001). Among donor livers heterozygous for the *CFH* H2 haplotype, a trend was observed indicating additional dose-dependent protection by the low-expressing *CFHR2* variant in Kaplan–Meier analyses, but this was not significant (Figure 4B, log-rank *p*=0.06). Therefore, *CFHR* variants may be associated with rejection risk in liver transplantation, beyond their linked *CFH* haplotype.

**FIGURE 4:**
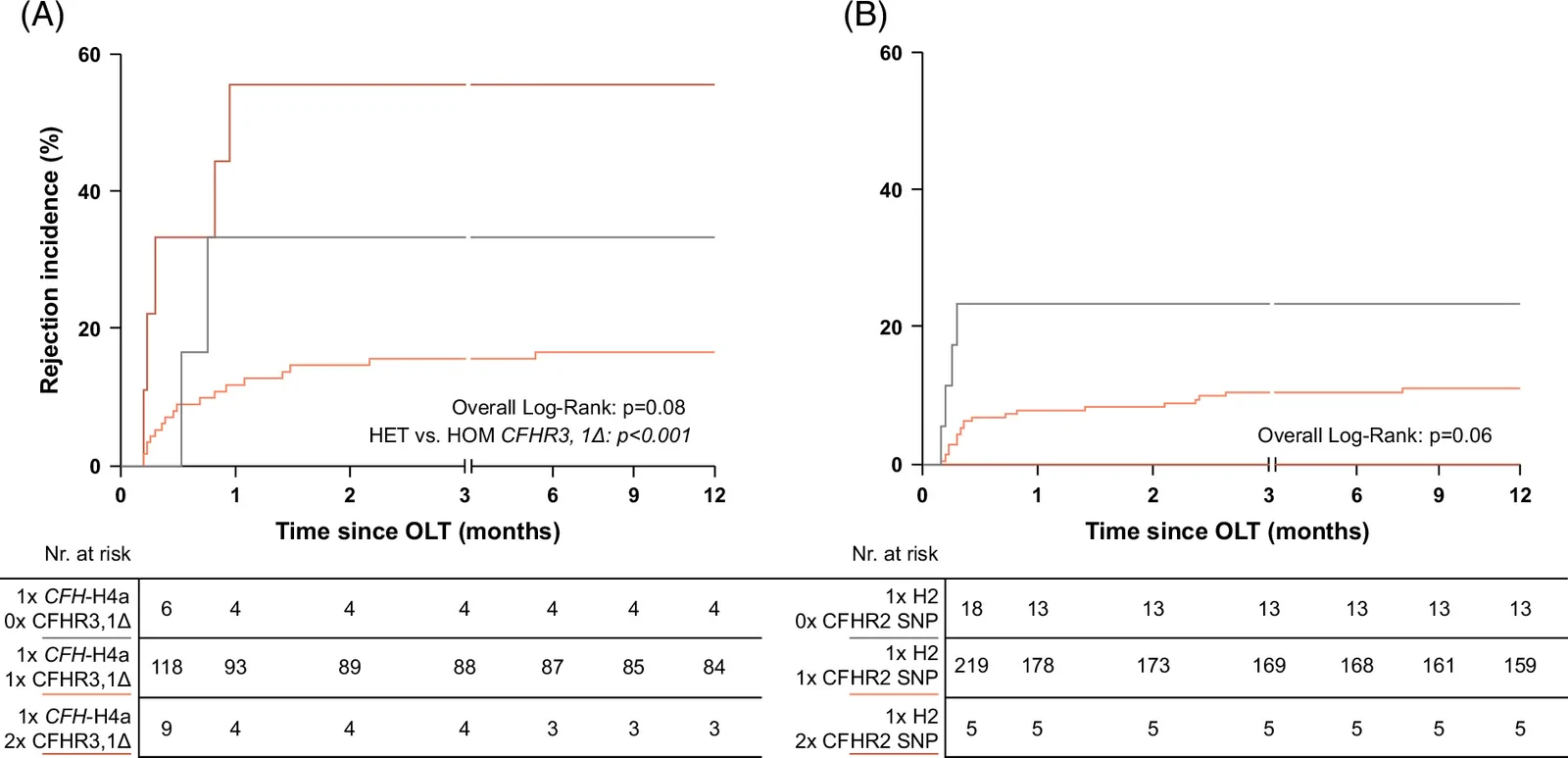
Kaplan–Meier cumulative risk curves depicting the effect of *CFHR* variants beyond their linked *CFH* haplotype. Kaplan–Meier cumulative risk curves, with log-rank testing, comparing ACR incidence between differing donor *CFHR3,1Δ* genotypes in *CFH*-H4a heterozygous donors (A) and differing donor *CFHR2* genotypes in *CFH*-H2 heterozygous donors (B). Abbreviations: ACR, acute T-cell–mediated rejection; *CFH*, complement factor H gene; *CFHR*, complement factor H-related gene; *CFHR3,1Δ*, deletion of *CFHR3* and *CFHR1*; OLT, orthotopic liver transplantation.

### External replication

To replicate the analysis of our Dutch cohort, we aimed to utilize the liver transplant cohorts from Baylor (N=720) and BioTIP (N=437) in the United States. Given that the frequency of SNPs within the *CFH*–*CFHRs* region varies significantly across geographical locations and ethnicities,^14^^,^^32^ we first analyzed the genotype frequency and linkage disequilibrium (LD) structure of the *CFH* SNPs needed for the haplotype analyses. Our results revealed a notably different LD structure in both American cohorts compared with the Dutch cohort, despite selecting only individuals of European descent in all cohorts (Figure 5). In both Baylor (Figure 5B) and BioTIP (Figure 5C), the LD was so high among nearly all *CFH* SNPs (*r* ^2^ of 1 between 8 SNP-to-SNP comparisons compared with just 1 comparison in the Dutch cohort) that individual *CFH* haplotypes could not be differentiated, rendering replication of the findings impossible. Consistent with this observation, analysis of the 1000 Genomes Project reference dataset revealed clear ancestry-related differences in the LD structure across the *CFH* locus (Supplemental Figure S4, http://links.lww.com/HC9/C389). Given the heterogeneity of the haplotype associations, we concluded that including an overall tagging SNP would not meaningfully contribute to this analysis.

**FIGURE 5:**
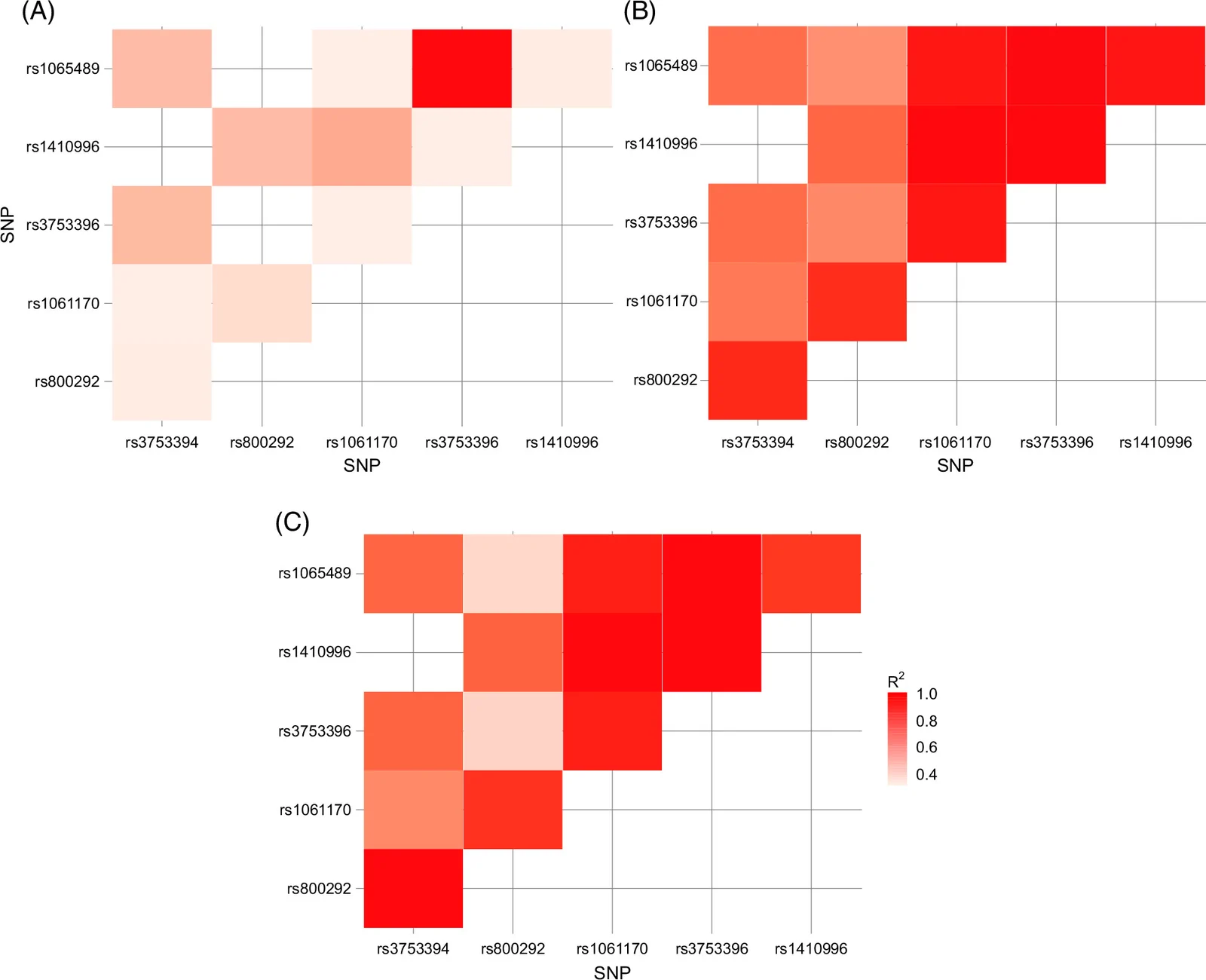
Differing linkage disequilibrium structures of *CFH* SNPs between Dutch and U.S. cohorts. Matrices depicting linkage disequilibrium (LD, *R* ^2^) between the 6 SNPs used to form the 9 distinct *CFH* haplotypes in donors and recipients of European descent from 3 separate cohorts. Panel (A) shows the LD in the Dutch cohort. Panel (B) shows the LD for donors and recipients from Baylor, a U.S. cohort. Panel (C) shows the LD for donors and recipients from BioTIP, a U.S. cohort. Abbreviations: *CFH*, complement factor H gene; LD, linkage disequilibrium; SNP, single-nucleotide polymorphism.

## DISCUSSION

Human genetics provides an opportunity to uncover the underlying mechanisms of pathology, ranging from metabolic disorders like type 2 diabetes and hypercholesterolemia to psychiatric conditions, and identify targets for therapeutic strategies.^33^ In this study, we found that common genetic variants in the *CFH–CFHR* region are significantly associated with ACR risk following liver transplantation. In particular, the *CFHR3,1Δ* in the donor and the recipient was associated with an increased risk of ACR. Furthermore, liver transplants from donors carrying the *CFH* H2 haplotype showed a reduced risk of developing ACR, whereas grafts from donors with the *CFH* H4a haplotype were associated with increased risk. To the best of our knowledge, this study is the first to offer genetic evidence for a key role of the factor H protein family in graft rejection after liver transplantation. Historically, research on complement in the field of liver transplantation has been limited, while complement genetics has been extensively studied in infections and inflammatory diseases. However, recent studies have utilized genetics to examine the role of the complement system in outcomes after a liver transplantation.^34^ In addition to genetic studies, research has identified complement proteins as early biomarkers of ACR.^35^ These findings collectively emphasize a potentially significant role of the complement system in liver transplantation.

The polymorphic nature of the complement system has been well documented for over 50 years.^36^ Common polymorphisms in complement genes have been associated with various pathologies, with *CFH* representing a major susceptibility locus.^12^^,^^13^ Functional studies of these disease-linked *CFH* variants have subsequently uncovered how they impact factor H’s function or expression, leading to changes in complement activity.^12^^,^^13^^,^^19^ Our analyses revealed a protective effect of the *CFH* H2 haplotype on ACR, which is consistent with prior studies showing its role in reducing complement activity and susceptibility to immune-mediated diseases. In AMD, large GWAS in European and Asian cohorts showed that carriers of the *CFH* H2 haplotype had a significantly lower disease risk.^24^^,^^37^ In aHUS and C3G, case-control and observational studies revealed that the same haplotype was underrepresented among affected individuals.^25^^,^^37^ Mechanistically, these findings indicate that an increased ratio of factor H to FHRs enhances complement regulation and thereby limits tissue injury and complement-mediated diseases. Additionally, we identified the *CFH* H4a haplotype as a risk factor for ACR. This haplotype has previously been associated with increased susceptibility to aHUS, where *CFHR3,1Δ* confers disease risk through its link with the development of factor H-blocking autoantibodies.^12^^,^^13^^,^^38^

Beyond the *CFH*, variations in the *CFHRs* have been associated with multiple diseases. *CFHRs* evolved through duplication events of the *CFH*, resulting in structural similarities to factor H while lacking its inhibitory region.^12^^,^^39^ In vitro studies support this concept, showing that FHRs can compete with factor H for ligand binding and thereby promote complement activation under certain conditions.^12^^,^^40^ At the same time, there is evidence that FHRs can bind to apoptotic and necrotic cells and act as opsonins, thereby promoting recognition and uptake by phagocytes.^41^ Functional studies of FHRs are still in an early stage, and only recently has it become possible to reliably measure FHR levels in human samples through the SciFiMed consortium.^42^ As a result, explanations of their role in disease and transplantation remain largely speculative. However, the general consensus from all proposed mechanisms is that reduced FHR levels shift the balance toward enhanced complement regulation, although the net effect is likely tissue- and context-specific.^12^

Amongst the disease-associated *CFHRs* variants, the *CFHR3,1Δ* is the most extensively studied and has been found to be protective against AMD and IgA nephropathy while conferring risk for aHUS and systemic lupus erythematosus.^12^^,^^13^^,^^15^^,^^16^ Mechanistically, *CFHR3,1Δ* leads to absent FHR-1 and FHR-3 levels and is associated with increased factor H levels. The *CFHR3,1Δ* is thought to confer protection in IgA nephropathy through enhanced factor H regulation and reduced C3 activation.^43^ In contrast, the same deletion increases aHUS risk via factor H autoantibodies, and similar mechanisms involving adaptive immune regulation and autoantibody production could explain the increased risk of systemic lupus erythematosus.^16^^,^^44^^,^^45^ Together, these findings underscore the context-dependent roles of FHRs in human disease. Regarding the *CFHR2* variant studied here, prior research has shown that it leads to reduced levels of total FHR-2, FHR-2 homodimers, and FHR-1/FHR-2 heterodimers.^18^ However, the direct impact of reduced FHR-1/FHR-2 heterodimers and FHR-2 homodimers on complement activity in specific disease contexts has not yet been investigated. What has been established, however, is that the *CFHR2*-variant-associated-*CFH*-H2 haplotype increases the ratio of factor H to FHRs,^18^^,^^46^ potentially enhancing complement regulation and limiting tissue injury.

In our liver transplant cohort, the presence of *CFHR3,1Δ* in either the donor or the recipient was a risk factor for ACR. This risk was further elevated when the deletion was present in both donor and recipient, with a hazard ratio of nearly 3.5 compared with pairs without the *CFHR3,1Δ*. The observation that the *CFHR3,1Δ* in both donor and recipient confers a higher ACR risk than its presence in either the donor or the recipient alone sheds new light on the interpretation of Markkinen et al's^17^ findings, which attributed the increased ACR risk to donor–recipient *CFHR3,1Δ* mismatch. Our results suggest that the observed association of Markkinen et al may instead reflect the impact of donor *CFHR3,1Δ* alone. In addition, our finding that recipient *CFHR3,1Δ* also influences ACR risk warrants further investigation into the potential non-hepatic production of FHRs. Taken together, our findings suggest that reduced FHR-1 and/or FHR-3 levels due to the combined deletion in the donor and/or recipient increase the risk of ACR. This also highlights that overall FHR availability, not just the presence of the deletion, is critical. When both donor and recipient carry the deletion, circulating FHR-1 and FHR-3 are absent, potentially impairing clearance of cellular debris and promoting adaptive immune activation. If only the donor carries the deletion, residual FHR-1 and FHR-3 from extrahepatic sources may partially preserve complement clearance. These gene-dose-dependent effects remain speculative. Further research is required to confirm our findings and to better understand the mechanisms linking *CFH–* *CFHR* genetic variation to the risk of ACR. Given our observation that recipient genotype influences rejection risk, future studies should investigate the potential extrahepatic production of these proteins. In addition, measuring circulating factor H and FHRs concentrations in liver transplant recipients will allow a more precise evaluation of the independent contribution of individual components to rejection risk.^42^ This is particularly relevant because the *CFH* haplotypes and *CFHR* variants examined in the present study have multiple effects on different members of the factor H protein family, and because substantial interindividual variation in factor H and FHRs levels exists even among individuals carrying the same haplotype.^19^ Finally, animal models will be essential to establish causality and provide mechanistic insight.^47^ For example, *Cfhr* ^*−/−*^ mice would offer a powerful system to investigate the role of FHRs in shaping B-cell and T-cell responses.

The *CFH* haplotypes are closely linked to genetic variations in the *CFHRs*,^12^^,^^13^^,^^19^^,^^31^^,^^39^ underscoring the importance of investigating *CFH* and *CFHR* together. Given the strong correlation between *CFH* H4a and *CFHR3,1Δ*, and between *CFH* H2 and the *CFHR2* nonsense variant, we explored whether these associations are solely driven by the *CFH* haplotypes or if the *CFHR* variants exert additional effects. Previous studies have indicated that the *CFHR* variants contribute additional risk, accounting for part of the disease associations beyond their respective *CFH* haplotype. Notably, Lorés-Motta et al^46^ demonstrated that *CFHR2* and *CFHR5* variants, which reduced their respective circulating levels, were independently associated with AMD risk after adjusting for multiple *CFH* variants. We hypothesize that FHRs play an FH-independent role in clearing cellular debris. In the absence of FHR-1 and FHR-3, necrotic and apoptotic cells accumulate following ischemia-reperfusion injury, leading to adaptive immune activation and, subsequently, triggering alloimmunity and rejection.^41^^,^^48^ Our analysis suggests that the *CFHR3,1Δ* exerts an additional harmful effect beyond the *CFH* H4a haplotype. Specifically, being homozygous for *CFHR3,1Δ* in the H4a-positive subgroup was associated with a higher ACR risk compared with being heterozygous. For the *CFHR2* nonsense variant, we could not confidently separate its effect from the *CFH* H2 haplotype on ACR. Given the small subgroup sizes, these findings should be considered preliminary and require validation in larger cohorts. Moreover, given the multiple functional effects of *CFH* haplotypes and the limitations of our genetic study, future research should assess factor H and FHRs plasma levels and the development of anti-factor H antibodies in donor–recipient pairs, and employ animal studies to examine the role of FHRs in B-cell and T-cell responses,^12^^,^^47^ thereby uncovering how FHRs may contribute to the development of ACR in liver transplantation.

We aimed to repeat our analyses in 2 independent cohorts to confirm our findings. However, despite the fact that both cohorts from the United States consisted of individuals of European ancestry, we observed much stronger LD between individual *CFH* SNPs of the haplotypes compared with our Dutch cohort. As a result, the *CFH* haplotypes were too closely associated in the U.S. cohorts to properly distinguish individual haplotype effects. Future studies, particularly when investigating relatively understudied populations, should examine LD structure before interpreting associations between *CFH* haplotypes and disease. Most studies linking *CFH* haplotypes to diseases have been conducted in European populations, and previous research has suggested that the effects of genetic variations in *CFH* may be weaker in non-European cohorts.^49^ We hypothesize that the U.S. cohorts in our study originated from a relatively small, specific ancestral group, leading to limited genetic variability. While differences in genotyping chips may have slightly affected the LD structure, the Affymetrix chip used in the U.S. cohorts provides extensive coverage of immune-related genes like *CFH*. Therefore, we believe the observed cohort differences are more likely biological rather than technical. Accordingly, our findings may not generalize beyond Northwestern European populations.

The findings of our study should be interpreted considering several limitations. First, the observational design precludes definitive conclusions about causality. In some analyses, we combined groups that ideally would have been evaluated separately (ie, heterozygous and homozygous *CFH* H4a haplotype). Statistical power was limited for certain *CFH*–*CFHR* analyses. For example, the analysis of *CFHR3,1Δ* in recipients with ACR may have been underpowered. Likewise, the study lacked sufficient power to perform subgroup analyses by donor type, sex, or other variables, limiting the exploration of their potential influence on the observed associations. Furthermore, although the association between *CFHR* genotypes and ACR remained significant after adjustment for transplant era in multivariable models as well as in subgroup analyses of more recent transplants, we acknowledge the relatively long inclusion period of our liver transplantation cohort. However, this limitation is inherent to genetic studies in liver transplantation due to the relative rarity of the procedure. Replication of our findings in larger, independent cohorts with comparable LD structures will therefore be essential before drawing definitive conclusions. Future research should also explore the impact of *CFH* and *CFHR* genetic variants on additional clinical outcomes beyond ACR, as well as the combined influence of other immunogenetic factors. Nonetheless, the strengths of our study lie in the comprehensive assessment of extended *CFH* haplotypes and a relatively large sample size for the liver transplantation field, supported by robust statistical analyses.

In conclusion, our study highlights a previously overlooked role of the factor H protein family in liver transplant rejection. A better understanding of the complement system’s role in rejection could potentially identify patients at increased ACR risk and tailor immunosuppression strategies to better suit individual patients. *CFH–CFHR* donor and recipient genotypes hold promise for future prediction of rejection risk and tailored immunosuppression, but current evidence is insufficient for clinical risk stratification. Still, these findings advance transplantation genetics and provide a foundation for larger, definitive studies. The results from our study, together with ongoing research, will hopefully bring us closer to a more personalized approach in liver transplantation, with the goal of improving long-term outcomes.

## Supplementary Material
