# Supplementary material for: Extended haplotypes at the complement factor H gene locus are associated with liver transplant rejection
Source: Hepatol Commun. 2026 Jul 15;10(8):e00997. doi: 10.1097/HC9.0000000000000997 (PMC13671603; doi:10.1097/HC9.0000000000000997)
Supplement: Supplementary file 1 [file hc9-10-e00997-s001.docx]

**Supplementary Figure legends**

**Supplementary Figure 1. the genomic locations of *CFH and CFHRs* genes, and the 9 distinct CFH haplotypes defined by six SNPs within *CFH***

Panel A illustrates the genomic locations of the *CFH* and *CFHR* genes. Panel B depicts the various combinations of alleles from six SNPs within CFH that form 9 distinct haplotypes. Panel C depicts the frequency (%) of each haplotype in the donor and recipient cohorts.

**Supplementary Figure 2. Kaplan-Meier cumulative risk curves for post-1996 cohort confirms associations of *CFHR3,1Δ* and *CFHR2* with ACR**

Kaplan-Meier cumulative risk curves, with log-rank testing, comparing ACR incidence between differing donor- (A), recipient- (B) *CFHR3,1Δ*-genotypes, and (C) donor *CFHR2* genotypes in the post-1996 cohort.

**Supplementary Figure 3. Venn diagrams depicting the overlap between *CFH*-H4a and *CFH*-H2 haplotypes with their corresponding *CFHR* variant**

Venn diagrams depicting the overlap between the *CFH*-H4a haplotype and *CFHR3,1Δ* genotypes in the donor (A) and recipient (B); and between the *CFH*-H2 haplotype and *CFHR2* genotypes in the donor (C) and recipient (D).

**Supplementary Figure 4. Ancestry-related differences in the linkage disequilibrium structure across the CFH locus**

Matrices depicting linkage disequilibrium (LD, R^2^) between the six SNPs used to form the 9 distinct *CFH* haplotypes humans from differing ancestries, using the 1000 genomes reference datasets. Panel A shows the LD in a cohort op people of Nigerian descent. Panel B shows the LD in a cohort op people of Vietnamese descent. Panel C shows the LD in a cohort op people of Italian descent. Panel D shows the LD in a cohort op people of Mexican descent.
