# Supplementary material for: Extended haplotypes at the complement factor H gene locus are associated with liver transplant rejection
Source: Hepatol Commun. 2026 Jul 15;10(8):e00997. doi: 10.1097/HC9.0000000000000997 (PMC13671603; doi:10.1097/HC9.0000000000000997)
Supplement: Supplementary file 2 [file hc9-10-e00997-s002.docx]

**Supplementary Table 1. Individual frequencies of CFH haplotype polymorphisms**

| **SNP** | | **Donor (N=689)**  *N* (%) | | **Recipient (N=689)**  *N* (%) | | *p*-value |
| --- | --- | --- | --- | --- | --- | --- |
| *CFH* rs3753394 | C\|C | 369 | 54% | 368 | 53% | 0.95 |
|  | C\|T | 264 | 58% | 268 | 39% |  |
|  | T\|T | 56 | 8% | 53 | 8% |  |
| *CFH* rs800292 | G\|G | 372 | 54% | 383 | 56% | 0.52 |
|  | G\|A | 259 | 37% | 259 | 38% |  |
|  | A\|A | 58 | 8% | 47 | 8% |  |
| *CFH* rs1061170 | C\|C | 97 | 14% | 104 | 15% | 0.25 |
|  | C\|T | 316 | 46% | 339 | 49% |  |
|  | T\|T | 276 | 40% | 246 | 36% |  |
| *CFH*  rs3753396 | A\|A | 487 | 71% | 483 | 70% | 0.74 |
|  | A\|G | 181 | 26% | 189 | 27% |  |
|  | G\|G | 21 | 3% | 17 | 3% |  |
| *CFH*  rs1410996 | G\|G | 230 | 33% | 242 | 35% | 0.20 |
|  | G\|A | 329 | 48% | 342 | 50% |  |
|  | A\|A | 130 | 19% | 105 | 15% |  |
| *CFH*  rs3024498 | G\|G | 486 | 71% | 485 | 70% | 0.69 |
|  | G\|T | 181 | 26% | 187 | 27% |  |
|  | T\|T | 22 | 3% | 17 | 3% |  |
| *Tag SNP CFHR3,1Δ*  rs6677604 | G\|G | 462 | 67% | 476 | 69% | 0.70 |
|  | G\|A | 205 | 30% | 191 | 28% |  |
|  | A\|A | 22 | 3% | 22 | 3% |  |
| *CFHR2*  rs4085749 | C\|C | 376 | 55% | 391 | 57% | 0.67 |
|  | C\|T | 266 | 39% | 250 | 36% |  |
|  | T\|T | 47 | 7% | 48 | 7% |  |

The prevalence of the genotypes per SNP were compared between the liver transplant recipients and their donors in the Dutch cohort. Data are displayed as the percentages of the parent population and *p*-values of the Pearson’s χ^2^ tests. *P*-value comparing liver transplant donors and recipients in our cohort.

*N*, number; *SNP*, single nucleotide polymorphism; *CFH*, complement factor H
