# Supplementary material for: Extended haplotypes at the complement factor H gene locus are associated with liver transplant rejection
Source: Hepatol Commun. 2026 Jul 15;10(8):e00997. doi: 10.1097/HC9.0000000000000997 (PMC13671603; doi:10.1097/HC9.0000000000000997)
Supplement: Supplementary file 3 [file hc9-10-e00997-s003.docx]

**Supplementary Table 2. No association between complement factor H haplotypes in the recipient and ACR**

| 90 rejection vs 599 controls | **Univariable** | | | |
| --- | --- | --- | --- | --- |
|  | HR | | 95% CI | P-value |
| H1 yes (no=ref)  H2  H3  H4a  H4b  H5-8 | 1.06  0.67  0.59  1.34  1.36  1.26 | | 0.70-1.62  0.44-1.07  0.34-1.01  0.82-2.18  0.77-2.40  0.72-2.190 | 0.78  0.10  0.06  0.24  0.29  0.42 |

Note: Univariable cox regression analyses were performed with ACR as a binary outcome (presence or absence). For each individual haplotype we set the reference as the absence (zero copies) of that haplotype, with presence of a haplotype meaning at least one copy of that haplotype. Since none of the haplotypes were significant in the univariable analyses, we did not perform any multivariable analyses. Data are presented as hazard ratios with confidence ratios and corresponding p-values. In the multivariable analysis we have corrected for Immune-mediated indications for OLT (PSC, PBC/AIH), CIT, recipient CMV status at OLT and time since transplantation.

Abbreviations: HR, hazard ratio; CI, confidence interval
