# Supplementary material for: Extended haplotypes at the complement factor H gene locus are associated with liver transplant rejection
Source: Hepatol Commun. 2026 Jul 15;10(8):e00997. doi: 10.1097/HC9.0000000000000997 (PMC13671603; doi:10.1097/HC9.0000000000000997)
Supplement: Supplementary file 4 [file hc9-10-e00997-s004.docx]

**Supplementary Table 3. No impact of HLA mismatch on adjusted associations between ACR and *CFHR3,1Δ* or *CFHR2* polymorphisms**

| 90 rejection  vs 599 controls | **Donor *CFHR3,1Δ - ACR*** | | | **Recipient *CFHR3,1Δ - ACR*** | | | **Donor *CFHR2* polymorphism - ACR** | | |
| --- | --- | --- | --- | --- | --- | --- | --- | --- | --- |
|  | HR | 95% CI | P-value | HR | 95% CI | P-value | HR | 95% CI | P-value |
| Not adjusted for HLA mismatch | 2.76 | 1.18-6.45 | **0.02** | 2.55 | 1.15-5.65 | **0.02** | 0.66 | 0.45-0.96 | **0.03** |
| Adjusted for HLA mismatch | 2.73 | 1.17-6.37 | **0.02** | 2.66 | 1.20-5.93 | **0.02** | 0.64 | 0.44-0.94 | **0.02** |

Note: Multivariable cox regression analyses were performed with ACR as a binary outcome (presence or absence). In both models time since transplantation (transplant era), immune-mediated transplant indications, recipient CMV status and cold ischemia time were included as confounders, with HLA mismatch added as a potential confounder in the second model. Data are presented as hazard ratios with confidence ratios and corresponding p-values.

Abbreviations: HLA, human leukocyte antigen; ACR, acute cellular rejection; CFHR3,1Δ, Complement Factor H-Related 3 and Complement Factor H-Related 1 Deletion; CFHR2, Complement Factor H-Related 2; HR, hazard ratio; CI, confidence interval; CIT, cold ischemia time
