# Supplementary material for: Extended haplotypes at the complement factor H gene locus are associated with liver transplant rejection
Source: Hepatol Commun. 2026 Jul 15;10(8):e00997. doi: 10.1097/HC9.0000000000000997 (PMC13671603; doi:10.1097/HC9.0000000000000997)
Supplement: Supplementary file 5 [file hc9-10-e00997-s005.docx]

Supplemental methods

*DNA extraction, genotyping, quality control and non-HLA imputation*

Blood for DNA extraction was collected at transplantation from donors and recipients. Genomic DNA was extracted from whole blood or peripheral blood mononuclear cells, using standard procedures and stored at -80 °C. DNA quality and concentration were assessed as previously described.^1^ In the Dutch cohort genotyping was performed using the Infinium Global Screening Array-24 v1.0 (Illumina Inc., San Diego, USA). In the replication cohorts genotyping was performed using the Affymetrix Axiom Transplant Array. Genotype data was clustered using the GenomeStudio software^2^, and quality control was performed using PLINK^3^. Normalized intensities for all samples were called using Opticall clustering^4^. Raw genotypes were imputed using the TOPMed reference panel (version r2, hg38).^5-7^ Post-imputation quality control was performed with an estimated imputation accuracy of at least 0.4.^8^

For the *CFHR3,1Δ*, we utilized the well-established tag SNPs rs6677604 (567520A>G) and rs7542235 (619531G>A).^9^ As both SNPs have similar MAF and near perfect linkage disequilibrium (LD) (D’: 0.99, r2: 0.96),^9^ we performed all analyses exclusively with rs6677604. Additionally, we included the nonsense SNP rs4085749 (c.C420T), located within *CFHR2,* which results in a near null allele.^10-12^ Both SNPs were extracted from the imputed genotype screening array dataset for all donors and recipients.

Six SNPs in *CFH* (rs3753394, rs800292, rs1061170, rs3753396, rs1410996, rs1065489) form distinct haplotypes (Supplementary **Fig. 1B**).^13^ To determine *CFH* haplotypes, we phased all SNPs within 1000kb of rs1061170 using SHAPEIT (version 2.r904)^14^ and then extracted the six SNPs from both donor and recipient genotypes. To replicate results from the *CFH* haplotype analyses, we compared the LD of the six *CFH* SNPs between the two replication cohorts and the Dutch cohort, solely in donors and recipients of European descent.

*HLA imputation and mismatch calculation*

HLA class I and II genotypes for all donor-recipient pairs were imputed using the HLA-dedicated imputation algorithm CookHLA (v1.0.1)^15^, using the reference panel from the Type 1 Diabetes Genetics Consortium^16^. Unphased PLINK GSA data (build hg18) were used as input, which was restricted to SNPs within the 29-34 Mb region of chromosome 6 (which encompasses the major histocompatibility complex region). HLA-Matchmaker^17^ was then used to calculate the combined donor-recipient mismatch of HLA-A, HLA-B, HLA-C, HLA-DRB1, HLA-DQB1, HLA-DQA1, HLA-DPB1, and HLA-DPA1 four-digit alleles.

References

1. **Nieuwenhuis LM, Li Y**, Loza BL, et al. Genome-wide meta-analysis associates donor-recipient non-HLA genetic mismatch with acute cellular rejection post-liver transplantation. *Hepatol Commun*. 2024 Dec 11;9(1):e0601. doi: 10.1097/HC9.0000000000000601. PMID: 39670865; PMCID: PMC11637756.
2. Illumina. Infinium ® Genotyping Data Analysis. *Tech Note*. 2014
3. Purcell S, Neale B, Todd-Brown K, et al. PLINK: a tool set for whole-genome association and population-based linkage analyses. *Am J Hum Genet*. 2007; doi:10.1086/519795
4. Shah TS, Liu JZ, Floyd JA, et al. OptiCall: A robust genotype-calling algorithm for rare, low-frequency and common variants. *Bioinformatics*. 2012; doi:10.1093/bioinformatics/bts180
5. **Taliun D, Harris DN, Kessler MD**, et al. Sequencing of 53,831 diverse genomes from the NHLBI TOPMed Program. *Biorxiv*. 2019; doi: 10.1038/s41586-021-03205-y
6. **Das S, Forer L, Schönherr S**, et al. Next-generation genotype imputation service and methods. *Nat Genet*. 2016; 48(10):1284-1287. doi:10.1038/ng.3656
7. Fuchsberger C, Abecasis GR, Hinds DA. minimac2: faster genotype imputation. *Bioinformatics*. 2014; 31(5), 782–784.
8. IC. A post-Imputation data checking program. Affymetrix Web site. Available from: <https://www.well.ox.ac.uk/~wrayner/tools/Post-Imputation.html>.
9. Dyer SC, Austine-Orimoloye O, Azov AG, et al. Ensembl 2025, *Nucleic Acids Research*, Volume 53, Issue D1, 6 January 2025, Pages D948–D957, <https://doi.org/10.1093/nar/gkae1071>
10. Lucientes-Continente L, Márquez-Tirado B, Goicoechea de Jorge E. The Factor H protein family: The switchers of the complement alternative pathway. *Immunol Rev*. 2023 Jan;313(1):25-45. doi: 10.1111/imr.13166. Epub 2022 Nov 16. PMID: 36382387; PMCID: PMC10099856.
11. Veuskens BRJ, Brouwer MC, Mierlo G, et al. Factor H related 2 levels dictate FHR dimer composition, 10 March 2025, PREPRINT (Version 1) available at Research Square [https://doi.org/10.21203/rs.3.rs-6130401/v1]
12. **Cipriani V, Lorés-Motta** L, He F, et al. Increased circulating levels of Factor H-Related Protein 4 are strongly associated with age-related macular degeneration. *Nat Commun*. 2020 Feb 7;11(1):778. doi: 10.1038/s41467-020-14499-3. PMID: 32034129; PMCID: PMC7005798.
13. de Córdoba SR, de Jorge EG. Translational mini-review series on complement factor H: genetics and disease associations of human complement factor H. *Clin Exp Immunol*. 2008 Jan;151(1):1-13. doi: 10.1111/j.1365-2249.2007.03552.x.
14. Delaneau O, Zagury JF, Robinson MR, Marchini J, Dermitzakis E. Accurate, scalable and integrative haplotype estimation. *Nat. Comm*. 2019. Doi: 10.1038/s41467-019-13225-y
15. Cook S, Choi W, Lim H, Luo Y, Kim K, Jia X, et al. CookHLA: Accurate imputation of human leukocyte antigens. Accessed March 26, 2023.
16. Rich S. Type 1 Diabetes Genetics Consortium (V7) [Dataset]. NIDDK Central Repository. 2023. 10.58020/qrdt-eh40
17. Duquesnoy RJ, Askar M. HLAMatchmaker: a molecularly based algorithm for histocompatibility determination. V. Eplet matching for HLA-DR, HLA-DQ, and HLA-DP. Hum Immunol. 2007;68:12–25.
